# The optimization of fermentation conditions for *Pichia pastoris* GS115 producing recombinant xylanase

**DOI:** 10.1002/elsc.201900116

**Published:** 2020-01-21

**Authors:** Taotao Sun, Ping Yan, Na Zhan, Licong Zhang, Zhihui Chen, Aizhong Zhang, Anshan Shan

**Affiliations:** ^1^ Laboratory of Molecular Nutrition and Immunity, The Institute of Animal Nutrition Northeast Agricultural University Harbin P. R. China; ^2^ College of Animal Science & Veterinary Medicine Heilongjiang Bayi Agricultural University Daqing P. R. China

**Keywords:** methodology optimization, Plackett–Burman, xylanase

## Abstract

Xylanase is a member of an important family of enzymes that has been used in many biotechnological processes. However, the overall cost of enzyme production has been the main problem in the industrial application of enzymes. To obtain maximum xylanase production, statistical approaches based on the Plackett–Burman design and response surface methodology were employed. The results of the statistical analyses demonstrated that the optimal conditions for increased xylanase production were the following: inoculum size, 3.8%; maize meal, 4.5%; histidine, 0.6%; methanol, 1%; culture volume, 20%; bean pulp, 30 g L^−1^; and Tween‐80, 0.8%; and pH 5.0. Verification of the optimization demonstrated that 3273 U mL^−1^ xylanase was observed under the optimal conditions in shake flask experiments. SDS–PAGE results showed that the size of xylanase protein was about 23 kDa. The results showed that the xylanase produced by fermentation came from *Aspergillus Niger* by MALDI‐TOF‐MS. The optimized medium resulted in 2.1‐ and 1.4‐fold higher the activity of xylanase compared with the unoptimized medium (the main nutrients are maize meal and bean pulp) and laboratory medium (the main nutrients are yeast extract and peptone), respectively. The optimization of fermentation conditions is an effective means to reduce production cost and improve xylanase activity.

**Abbreviations**
PBS: phosphate‐buffered saline
RSM: response surface method

## 1 INTRODUCTION

Xylan is one of the most important and representative hemicellulose resources, and is the second largest recyclable resource after cellulose 1. The hydrolysis reaction of hemicellulose is completed by the synergistic action of hemicellulases, including endo‐1,4‐β‐xylanase (E.C.3.2.1.8), xylan‐1,4‐β‐xylosidase (E.C.3.2.1.37), α‐glucosiduronase (E.C.3.2.1.139), α‐l‐arabinofuranosidase (E.C.3.2.1.55), and acetyl xylose esterase (E.C.3.1.1.72). The main component of hemicellulose in plant cell walls is xylan, which accounts for 35% of the dry weight of plant cells and is a rich biomass resource 2, 3. However, a large portion of the xylan in nature is not effectively utilized, resulting in a great waste of resources. In recent years, xylanases have been widely considered due to their great potential for industrial applications, low toxicity, and ability to reduce pollution such as pulp bleaching, bioethanol production, oligosaccharide production, and the processing of agricultural waste 4, 5, 6. Xylanases are widely used to improve growth performance, microbial counts, and the expression of inflammation in animal feeds 7, 8, 9, 10, 11.

Although xylanases have been purified and characterized, their activity is very low. The major obstacles to the wide‐range application of xylanases in industry are high cost and low activity. Pure substrates are very expensive, limiting the production of large quantities of enzymes at the industrial level 12, 13, 14. To reduce fermentation costs, maize meal and bean pulp, a type of cheap submerged fermentation substrate for the production of xylanase, are used as alternative substrates in this study. Cheap submerged fermentation substrates agroresidues are also cheap carbon source for solid state fermentation. Because the production of xylanase is strongly influenced by its culture conditions and fermentation (submerged or solid) medium composition, its optimization is very important to maintain the balance between process conditions and reduce the number of unutilized components.

The one‐factor‐at‐a‐time method is an experimental method that keeps all the conditions fixed at the same time a single factor is changed to study the phenomenon of interest. However, the main disadvantage of this method is that it ignores the interactions between two components 15, 16. In contrast, optimization with statistical methods, such as the response surface method (RSM) can quickly screen out large experimental domains and model the interaction of variables in the process; hence, each reaction parameter can be optimized in unison with other parameters to achieve maximum enzyme activity 17, 18. The RSM, the design of the experimental technology, can be used to establish mathematical models that can evaluate the effects of multiple factors on the expected response 19. The RSM involves the use of appropriate experimental quantitative data to identify and solve multivariable equations at the same time and is a statistical method that provides the best solution 20, 21. The RSM has the advantages of being convenient and saving time because it significantly reduces the evaluation of multiple parameters and their interactions, which would require a required number of experiments 22. The RSM is also a method for optimizing medium composition and other key variables 14, 22, 23, 24. In addition, multivariable experiments in the RSM reduce the number of optimizations required and provide more accurate results than single‐variable strategies, resulting in significant improvements in enzyme production 23. Therefore, the optimization of fermentation process parameters and medium components by statistical approaches, such as the Plackett–Burman design and the RSM, has been well appreciated for its significant improvements in activity and reduction in enzyme production costs 25, 26, 27, 28. Therefore, the culture conditions of high recombinant xylanase under deep fermentation conditions were optimized by statistical experimental design. Then, the response surface methodology combined with the Box–Behnken statistical design was used to further optimize the fermentation conditions; specifically, the percentage of inoculum size, maize meal, and histidine.

**PRACTICAL APPLICATION**

The high activity of xylanase showed that the application of xylanase in feed was suitable. Large‐scale fermentation is feasible in industry because of its cheap cost.

**Highlights**

- Successful application of maize meal and bean pulp as carbon and nitrogen sources, respectively, in the fermentation of GS115/pPICZa‐(XynB‐opt)_2_.
- Statistical approaches based on the Plackett–Burman design and response surface methodology were employed.
- The optimized medium resulted in 2.1‐ and 1.4‐fold higher the activity compared with the unoptimized medium (the main nutrients are maize meal and bean pulp) and laboratory medium (the main nutrients are yeast extract and peptone), respectively.

## 2 MATERIALS AND METHODS

### 2.1 Microorganism

*Pichia pastoris* GS115 with the vector pPICZa carrying xylanase‐encoding gene‐(XynB‐opt)_2_ was preserved in our laboratory 1. Briefly, the XynB gene encoding a mature xylanase of 225 amino was subcloned into the pPICZαA vector under the control of the alcohol oxidase I (AOX1) promoter and transformed into *P. pastoris* GS115 1, 29.

### 2.2 Seed culture and fermentation medium preparation

The seed culture medium contained the following substances: glucose, 20 g L^−1^; peptone, 20 g L^−1^; and yeast extract, 10 g L^−1^. The medium for xylanase production comprised NH_4_H_2_PO_4_, 25 g L^−1^; CaSO_4_, 0.5 g L^−1^; K_2_SO_4_, 9.1 g L^−1^; MgSO_4_, 7.5 g L^−1^; KOH, 1.5 g L^−1^; maize meal, 20 g L^−1^; and bean pulp, 20 g L^−1^. The substrate concentration and pH were adjusted and based on the experimental design. Then, the culture media were autoclaved at 115°C for 15 min.

### 2.3 Culture conditions

The microorganisms were cultured in 250 mL Erlenmeyer flasks containing 100 mL of the medium. A total of 2.5% inocula was inoculated in the medium. The flasks were incubated at 28°C, for 72 h, at 220 rpm in a rotary incubator shaker. At the end of fermentation, crude enzymes were obtained by centrifuging the supernatant fraction at 10 000 × *g* for 5 min.

## 3 EXPERIMENTAL DESIGN

### 3.1 Screening of factors affecting xylanase production

Experiments were conducted to screen the optimal process parameters and medium components by using the traditional one‐factor‐at‐a‐time technique. Variables were fixed at a certain value while only one variable value was changed (methanol, 0.5%; culture volume, 100 mL; inoculum size, 1%; maize meal, 20 g L^−1^; bean pulp, 20 g L^−1^; and histidine, 0.5%; pH 5.5). Each factor examined for optimization was included in the subsequent experiments. All other experimental conditions were kept constant unless otherwise stated.

### 3.2 Optimization of xylanase production using the Plackett–Burman design

First, the Plackett–Burman design was applied to screen important factors for enzyme production among eight variables, including methanol, culture volume, inocula size, initial pH, maize meal, bean pulp, Tween‐80, and histidine. Based on the Plackett–Burman factorial design, each factor was examined at the following two levels: −1 for low level and +1 for high level 18. The Plackett–Burman design was used in Design Expert 7.0 software to evaluate the eight factors. A xylanase activity assay was performed in triplicate and the average of these experimental values was taken as response Y. Then, based on the results of the Plackett–Burman design (*p* < 0.05) (Table 1), the method of steepest ascent was used to find the optimal regions of the important variables by increasing the values of the positive variable and reducing those of the negative variable.

**Table 1:** Plackett–Burman test design and results

Run no.	Methanol (%)	Culture volume (mL)	Inoculum size (%)	Initial pH	Maize meal (g L^−1^)	Bean pulp (g L^−1^)	Tween‐80 (%)	Histidine (%)	(*Y*) Enzyme activity (U mL^−1^)
1	1.5	55	4	5	40	20	10	3	2276.0
2	0.5	55	1	5	60	20	6	7	1874.0
3	1.5	45	4	4	60	40	6	3	2167.2
4	0.5	55	4	5	60	40	6	3	2268.4
5	0.5	45	1	5	40	40	10	3	2104.5
6	0.5	45	4	4	60	20	10	7	2039.6
7	1.5	45	1	5	60	40	10	7	1798.0
8	1.5	55	1	4	40	40	6	7	1982.4
9	1.5	55	1	4	60	20	10	3	2058.2
10	0.5	55	4	4	40	40	10	7	2272.7
11	1.5	45	4	5	40	20	6	7	2190.3
12	0.5	45	1	4	40	20	6	3	2173.8

The model equation for xylanase activity can be written as: *Y* = *β*0 + ∑*βi χi*, where *Y* is xylanase activity, *β*0 is intercept of the mode, *βi* is linear coefficient of the model, and *χi* is level of independent variables.

### 3.3 Statistical optimization of xylanase production using the RSM

In this study, a Box–Behnken design was designed to study the empirical relationship between xylanase production and three potentially significant factors, namely, A, inoculum size (V/V); B, maize meal; and C, histidine. As shown in Table 7, each variable was attributed to three coded levels (1, 0, and −1). The other factor levels were kept at their optimum level. The Box–Behnken design was used in the Design Expert 7.0 software to evaluate the three factors. Each of these three factors had three coded levels consisting of 17 experimental runs for analyzing the experimental data, including five replicates at the center point 30, 31 (Table 3).

### 3.4 Purification of xylanase

The purification of the xylanase protein basically followed the method of previous work 32. In short, the fermentation supernatant was produced by centrifugation at 5000 rpm at 4°C for 10 min, then ammonium was added to the supernatant to 70% saturated, causing the enzyme to precipitate. By using saturated ammonium sulfate at 4°C for 30 min, the protein was precipitated from the supernatant. Then, the precipitate was resuspended in 50 mM phosphate‐buffered saline (PBS) and dialyzed overnight at 4°C using Snake Skin™ Pleated Dialysis Tubing (Pierce, USA) with the same buffer. Dialy‐sate was loaded onto a gel filtration column (Superdex™ 75 10/300 GL, 10 × 300 mm, GE, USA) equilibrated with PBS buffer 1, 33. Proteins were eluted from the column with PBS at a flow rate of 0.4 mL min^−1^. Active fractions were pooled for further studies.

### 3.5 Protein electrophoresis analysis and enzyme activity assays

To identify the purified enzyme, SDS–PAGE was carried out, and the gel stained with Coomassie Brilliant Blue R‐250. Birchwood xylan (Sigma, St. Louis, USA) was used as the substrate to measure xylanase activity. The amount of reducing sugars released from xylan (1% w/v in citrate phosphate buffer; pH 5.0) was measured at 50°C for 20 min to determine xylanase activity 32. One unit of xylanase activity was defined as the amount of enzyme that released 1 µmol of reducing sugar per minute from a substrate solution.

### 3.6 Statistical analysis

Design Expert 7.0 software was used for statistical analysis. All experiments were performed in triplicates. A significance level of *p* < 0.05 was considered significant. One‐way ANOVA showed no significant difference in the results of three repeated analyses. The *F*‐test was used to evaluate the significance of the regression coefficients. Regression coefficients were used for statistical calculation to generate dimensional and contour maps from the regression models.

## 4 RESULTS

### 4.1 Screening of medium components with a one‐factor‐at‐a‐time approach

**Figure 1:**
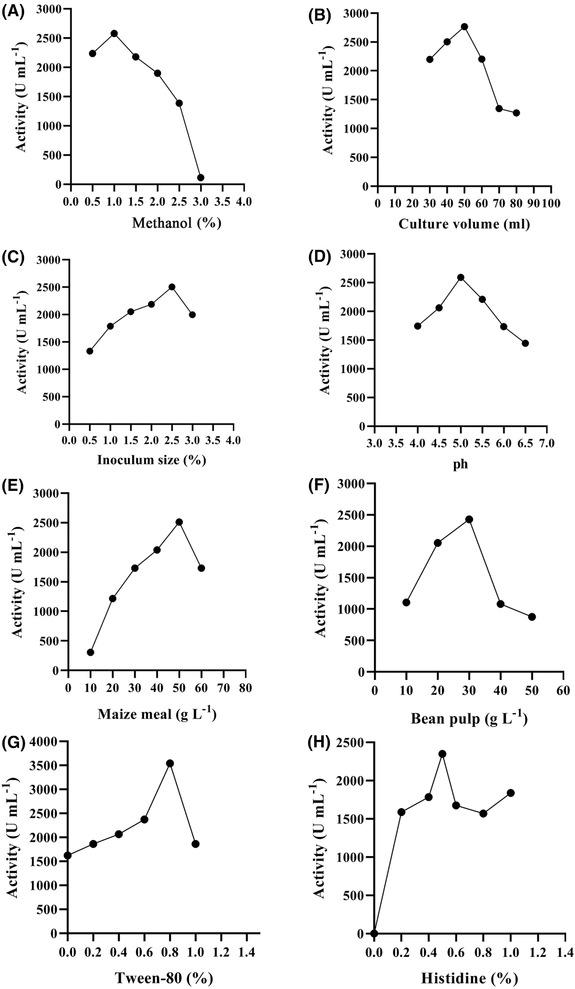
Effects of methanol (A), culture volume (B), inoculum size (C), pH (D), maize meal (E), bean pulp (F), Tween‐80 (G), and histidine (H) on xylanase activity

All variables were fixed at a certain value while only one variable was changed at a time (methanol, 0.5%; culture volume, 100 mL; inoculum size, 1%; maize meal, 20 g L^−1^; bean pulp, 20 g L^−1^; and histidine, 0.5%; pH 5.5). The eight factors were screened by testing the xylanase activity. As shown in Figure 1, the results revealed that the xylanase activity was maximum with the following parameters: methanol, 1%; culture volume, 20% (250 mL Erlenmeyer flasks containing 50 mL of the medium); inoculum size, 2.5%; maize meal, 50 g L^−1^; bean pulp, 30 g L^−1^; Tween‐80, 0.8%; and histidine, 0.5%; pH 5.0). Production decreased when the values of any of the factors were changed.

### 4.2 Evaluation of factors affecting xylanase enzyme production using the Plackett–Burman design

In the Plackett**–**Burman model, 12 experiments were needed as minimum tests to screen the importance of the eight medium components derived from the single factor optimization of xylanase. The screening result demonstrated that the xylanase activity varied widely ranging from 1798.0 U mL^−1^ to 2276.0 U mL^−1^ in 12 trials (Table 1).

Based on the calculated *p*‐values (Table 2), the analysis of regression coefficients and the *p*‐values of the eight parameters showed that inoculum size (V/V), maize meal (g L^−1^), and histidine (g L^−1^) stimulated xylanase production significantly (*p* < 0.05), while the other variables tested did not significantly influence xylanase activity (*p* > 0.05). Therefore, the inoculum size, maize meal, and histidine were selected for further optimization by the Box–Behnken design, and the other variables were kept at intermediate levels.

**Table 2:** Statistical analysis of the Plackett–Burman design showing sum of squares, mean square *F*‐value and *p*‐value for each variable for xylanase activity (*p*‐value < 0.05) (*R* ^2^ = 96.23%)

	Sum of squares	Mean square	*F*‐Value	*p*‐Value
Model	62.04	7.76	9.57	0.0449
Methanl (%)	1.39	1.39	1.71	0.2820
Culture volume (mL)	1.20	1.20	1.49	0.3100
Inoculm size % (V/V)	29.25	29.25	36.09	0.0092
Initial pH	0.93	0.93	1.14	0.3634
Maize meal (g L^−1^)	12.75	12.75	15.73	0.0286
Bean pulp (g L^−1^)	0.024	0.024	0.03	0.8738
Tween‐80 (%)	0.27	0.27	0.33	0.6057
Histidine % (g L^−1^)	16.22	16.22	20.01	0.0208

### 4.3 Response surface methodology

According to the previous Plackett**—**Burman screening test, the optimal conditions of the inoculum size, maize meal, and histidine were determined by the Box–Behnken design. A three‐factor and three‐level second‐order regression of the Box–Behnken design consisting of 17 experimental runs was employed including five replicates at the center point 30, 31 (Table 3).

**Table 3:** Design proposal and experimental results of the RSM

Run no.	Inoculum size	Maize meal	Histidine	Xylanase activity (U mL^−1^)
1	−1	−1	0	2070.7
2	1	−1	0	3061.0
3	−1	1	0	2160.7
4	1	1	0	2360.8
5	−1	0	−1	2320.8
6	1	0	−1	3071.0
7	−1	0	1	2570.9
8	1	0	1	3121.1
9	0	−1	−1	2720.9
10	0	1	−1	2550.9
11	0	−1	1	2901.0
12	0	1	1	2750.9
13	0	0	0	2960.0
14	0	0	0	2971.0
15	0	0	0	2920.5
16	0	0	0	2991.0
17	0	0	0	2930.0

The experimental design to determine the xylanase activity with Design Expert 7.0 software is shown in Table 3. With the results of these experiments, the second‐order model for xylanase activity was obtained in Equations (1) and (2) in coded and actual forms, respectively:

(1) $$\begin{array}{ccc} & & \text{R1:}\;\text{Xylanase}\;\text{enzyme}\\ & & \;=+2954.00+311.25\times A-116.25\times B+85.00\times C\\ & & \;-197.50\times A\times B-50.00\times A\times C+5.00\times B\times C\\ & & \;-\;250.75\times A2-290.75\times B2+66.75\times C2 \end{array}$$

(2) $$\begin{array}{ccc} & & \text{R1:}\;\text{Xylanase}\;\text{enzyme}\\ & & \;=-6530.37+1506.265\times A+3106.65\times B-958.45\times C\\ & & \;-\;131.70\times A\times B-166.67\times A\times C+24.88\times B\times C\\ & & \;-\;111.37\times A2-290.6\times B2+1675.9\times C2 \end{array}$$

**Figure 2:**
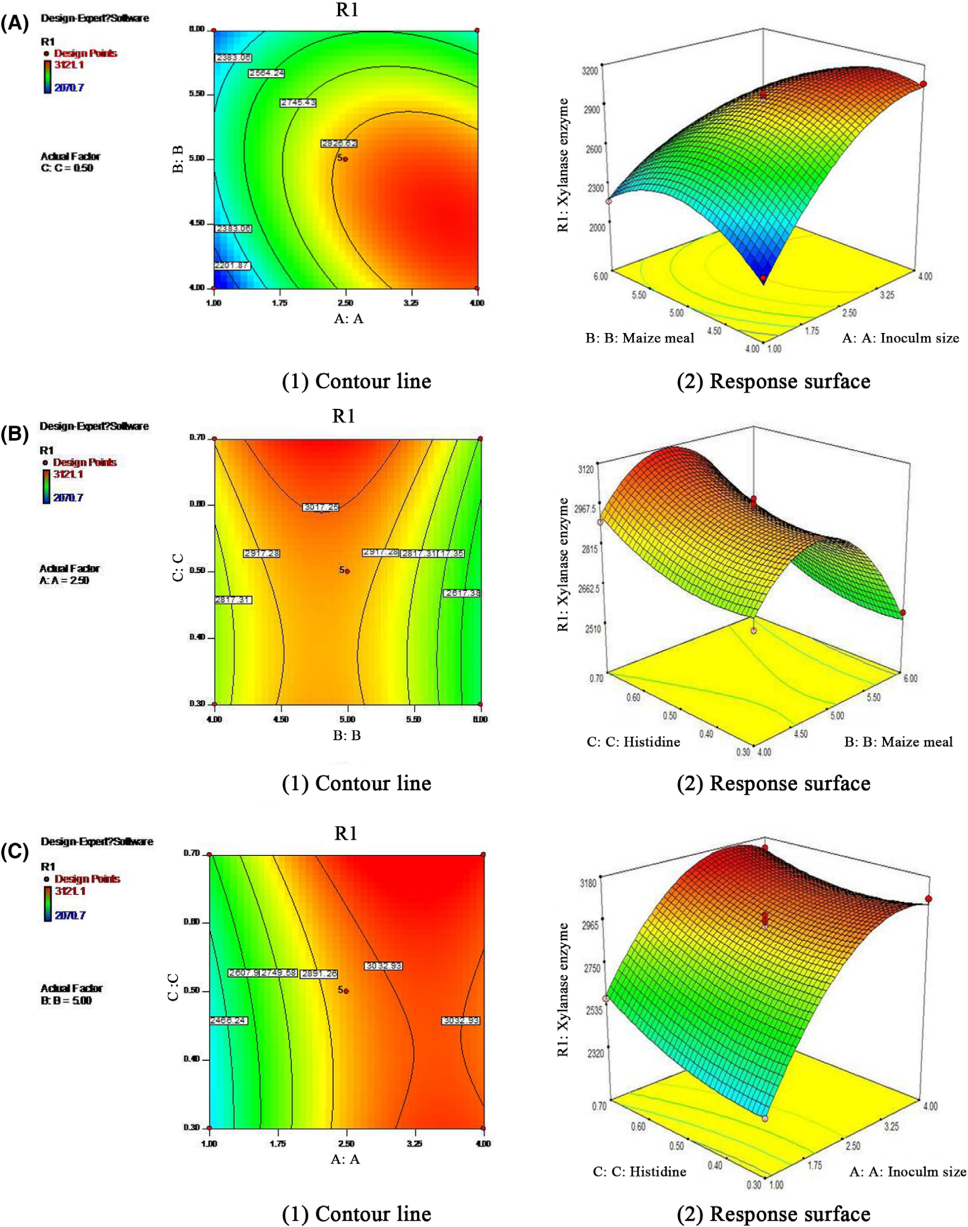
Response surface plots showing the effect of the selected parameters; inoculum size and maize meal (A), maize meal and histidine (B), and inoculum size and histidine (C)

As shown in Table 4, the *p*‐values of A‐A, inoculum size <0.0001; B‐B, maize meal = 0.0002; and C‐C, histidine = 0.0016 were all significant, suggesting that the three factors greatly influenced xylanase activity, and small changes in their respective values would markedly affect enzyme synthesis 34. The cross product coefficient AB was found to be significant, indicating a strong interaction between the inoculum size and maize meal. The other term coefficients (AC, BC) were not significant (*p* > 0.05). The variance analysis of the linear regression model showed that the model had high significance and that the *p*‐value was very low (*p* < 0.0001). Lack of fit, models failed to represent experimental data in the experimental field at points not included in regression analysis 35. A lack of fit was found to be nonsignificant (*p* = 0.0736), indicating that the model equation was sufficient to predict xylanase activity under any combination of the variables. The *p*‐value was a tool used to check the significance of each coefficient, and the smaller the *p*‐value, the more significant the corresponding coefficient is 30. Figure 2A–C shows response surface plots the effect of the selected parameters.

**Table 4:** ANOVA for response surface quadratic model for xylanase production

Source	Sum of squares	Mean square	*F*‐value	*p *> *F*	
Model	1.768 × 10^6^	1.964 × 10^5^	84.75	<0.0001	Significant
A‐A:Inoculum size	7.750 × 10^5^	7.750 × 10^5^	334.66	<0.0001	
B‐B:Maize meal	1.082 × 10^5^	1.082 × 10^5^	46.68	0.0002	
C‐C:Histidine	57851.01	57851.01	24.96	0.0016	
AB	1.561 × 10^5^	1.561 × 10^5^	67.36	<0.0001	
AC	10 000.00	10 000.00	4.32	0.0758	
BC	99.00	99.00	0.043	0.8421	
Residual	1 6221.25	2317.32			
Lack of Fit	1 2830.25	4276.75		0.0736	Not significant
Pure Error	3320.00	830.00			
*R^2^*	0.9909				
*R* ^2^Adj	0.9793				

The determination coefficient (*R* ^2^ = 0.9909) represented by the ANOVA of the quadratic regression model indicated that the model was highly significant and sufficient to predict in the range of experimental variables. The adjusted coefficient of determination (adjusted *R* ^2^ = 0.9793) was also high, which signified the high significance of the model (Table 4). Some researchers have reported a good correlation between experimental results and the Box–Behnken model predictions. From the above results, it could be concluded that the model could successfully predict the activity of xylanase and the coefficients obtained could be the direction of the steepest ascent method.

### 4.4 Validation of the model

**Figure 3:**
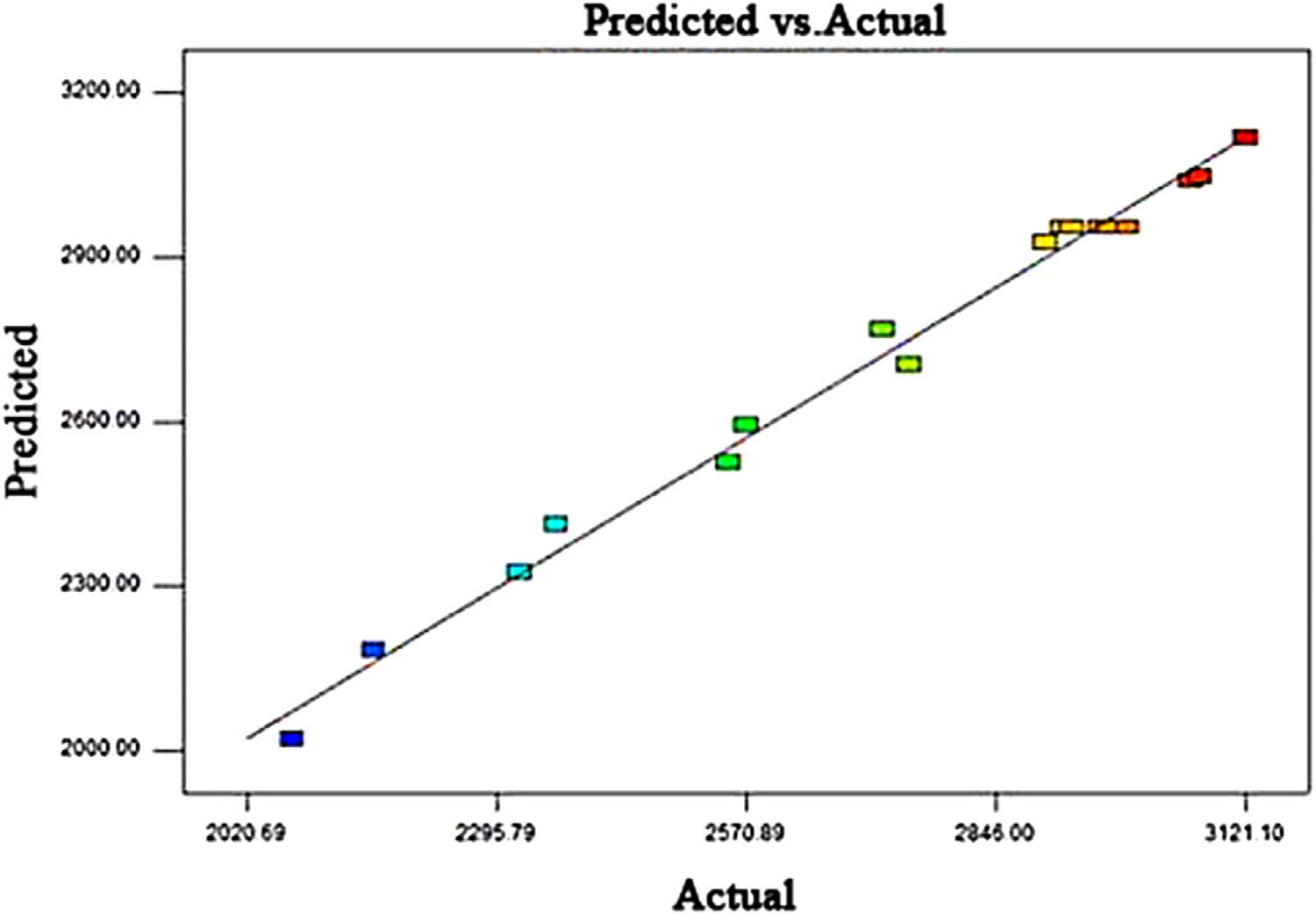
The relationship between the actual and predicted values for xylanase activity

The predicted conditions were verified by experimenting with the model prediction values. As shown in Figure 3, the results indicated that the model was successfully validated. These validation experiments suggested that inoculum size, 3.84%; maize meal, 4.61%; and histidine, 0.58% were optimal conditions for xylanase activity. Under these conditions, the model predicted the xylanase activity to be 3236.12 U mL^−1^. The results were optimized: inoculum size, 3.8%; maize meal, 4.5%; and histidine, 0.6%. The optimization validation results showed that the xylanase activity was 3273 U mL^−1^ under the optimal conditions in shake flask experiments. Hence, the RSM model based on Box–Behnken for predicting of the xylanase activity was accurate and reliable.

### 4.5 Purification and identification of xylanase

**Figure 4:**
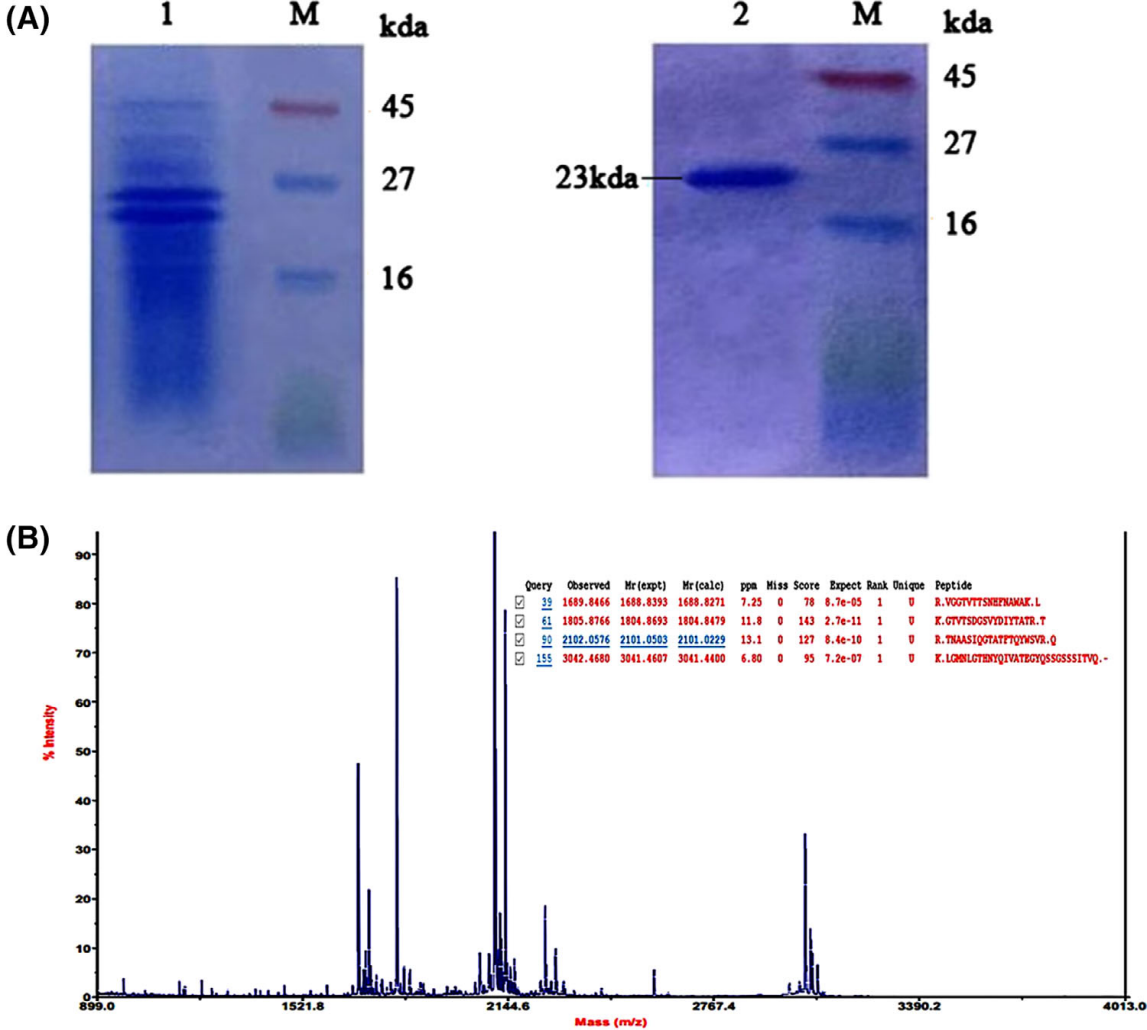
(A) Analysis of (XynB‐opt)_2_ after purification. 12% SDS–PAGE gels 1 and 2 loaded with 50 µg and 15 µg proteins, respectively. Lane M, protein standard; 1, supernatant of GS115/pPICZa‐(XynB‐opt)_2_ induced for 72 h; 2, purification of (XynB‐opt)_2_. (B) MALDI‐TOF‐MS analysis of GS115/pPICZa‐(XynB‐opt)_2_ and the matching enzyme

Xylanase was purified with SnakeSkin Pleated Dialysis Tubing. The recombinant enzyme was evaluated by SDS–PAGE analysis (Figure 4A) 36, 37, 38. The highly purified xylanase showed a single band on the Coomassie Brilliant Blue‐stained gel with a protein size of 23 kDa. According to the relative quantitative analysis of Figure 4A‐2, xylanase accounted for about 28.42% of the total secretory proteins before purification and 98.55% of the total secretory proteins after purification in using the software Alliance 4.7. The activity of xylanase before and after purification were 3273 U mL^−1^ and 2975 U mL^−1^, respectively. After purification, the specific activity of xylanase was 5926 U mg^−1^, and the yield of xylanase was 0.5020 g L^−1^.

To identify the protein, we cut out the second band (Figure 4A‐2). MALDI—TOF mass spectrum analysis is currently the most widespread protein appraisal method. GPS Explorer V3.6 software (Applied Biosystems, MA, USA) with default parameters was used to integrate and process the MS and MS/MS data. Based on the combined spectra of MS and MS/MS spectra, the proteins were successfully identified based on the 95% or higher confidence interval of their scores in the MASCOT V2.3 search engine (Matrix Science Ltd, London, UK) using the following search parameters: NCBI‐*Aspergillus niger* database; GI number of 13242071; Score of 442; Nominal mass (Mr) of 11059; Calculated pI of 8.18; and Sequence coverage of 78%. The results showed that the produced protein was xylanase from *A. niger*.

### 4.6 Characterization of (XynB‐opt)_2_ catalytic products

The mode of action of (XynB‐opt)_2_ was measured by using different xylo‐oligosaccharides (X2‐X6). (XynB‐opt)_2_ could hydrolyze X2–X6 and had little hydrolysis activity on xylobiose (X2). X2 was the minimum oligomer hydrolyzed by (XynB‐opt)_2_. The presence of relatively high concentrations of X2 in the xylooligosaccharides (X2–X6) indicated that the enzyme preferentially cleaved the internal glycosidic bonds of the above xylooligosaccharides (Table 5). Thus, it was concluded that (XynB‐opt)_2_ is a typical endo‐β‐1,4‐xylanase.

**Table 5:** IC analysis of the hydrolysis producers of X1‐based materials by (XynB‐opt)_2_

	Composition (%) of products formed by hydrolysis reaction				
Substrate	*X*1	*X*2	*X*3	*X*4	*X*5
X2	2.35%	97.65%	N^a^	N^a^	N^a^
X3	32.97%	39.90%	27.13%	N^a^	N^a^
X4	19.36%	64.30%	11.70%	4.64%	N^a^
X5	25.66%	56.95%	15.22%	2.17%	N^a^
X6	22.28%	66.83%	10.36%	0.53%	N^a^

*X*1 = xylose; *X*2 = xylobiose; *X*3 = xylotriose; *X*4 = xylotetraose; *X*5 = xylopentaose; *X*6 = xylohexasaccharide.

N^a^ = any products were not detected.

Analysis of the degradation products of soluble xylan (oat xylan, wheat xylan, and barley xylan) indicated that xylose was the major end product. Insoluble xylan (corncob xylan, bagasse xylan, birchwood xylan, and beechwood xylan) indicated that xylobiose was the major end product (Table 6).

**Table 6:** Xylan hydrolytic products concentration (%)

	Percentage content of hydrolysate			
Substrate	*X*1 (%)	*X*2 (%)	*X*3	*X*4
Oat xylan	98.38	1.62	N^a^	N^a^
Wheat xylan	22.13	77.75	0.12%	N^a^
Barley xylan	23.20	76.70	0.10%	N^a^
Corncob xylan	6.69	88.95	4.36%	N^a^
Bagasse xylan	4.78	91.34	3.88%	N^a^
Birchwood xylan	3.51	91.68	4.84%	N^a^
Beechwood xylan	2.97	90.57	6.46%	N^a^

Hydrolysis of different xylans by (XynB‐opt)_2_. Xylan (50 mg) was incubated with 3273 IU of the enzyme in 5 mL of Na_2_HPO_4_—citric acid buffer (50 mM, pH = 5.0), and the reaction was carried out at 40°C for 12 h; then, the hydrolysates were analyzed by IC.

N^a^ = Any products were not detected.

**Table 7:** Code of factors and levels

			Actual factors levels		
			at coded factor levels		
Variables	Units	Symbol	−1	0	1
Inoculum size	%	A	1.0	2.5	4.0
Maize meal	%	B	4.0	5.0	6.0
Histidine	%	C	0.3	0.5	0.7

## 5 DISCUSSION

Xylanase is widely used in industrial applications due to its potential application value. However, due to the high cost and low enzyme activity, the application of xylanase is limited 39, 40. The reduction in the cost of xylanase production is mainly through increasing the expression of xylanase and finding a cheaper and convenient fermentation medium 3, 14. In the past, the components of culture medium were usually yeast extract and peptone, which was not suitable for large scale fermentation in industry because of their high cost 1. So, it is necessary to choose cheap nutrients maize meal and bean pulp for fermentation 1, 41. Therefore, increasing the expression level of xylanase and reducing the cost have become key challenges in xylanase production 42. Thus, maize meal and bean pulp as cheap nutrients were chosen for the fermentation.

As shown in Figure 1C,H, a peak appeared when the inoculum size was 2.5% and the histidine value was 0.5%. Under this condition, the activity of xylanase was the highest. When the inoculation amount was relatively low, the content of the strain in the fermentation medium was insufficient, resulting in insufficient utilization of the medium and a decrease in expression. Since *P. pastoris* is an aerobic methanol nutritional yeast, dissolved oxygen in shake flask culture is a key factor that restricts cell growth and foreign protein expression. The amount of liquid in the shake flask was inversely proportional to the dissolved oxygen. However, when the volume of the shake flask was too low, the volatilization of the fermentation broth would increased with the fermentation time, causing the bacteria to stick to the wall. When the inoculation amount was too high, the content of the strain in the fermentation medium was too high, resulting in insufficient dissolved oxygen, an imbalance or insufficient nutrition, which affected the growth of the strain and led to low expression levels. The decline in inoculation yields was most likely due to anaerobic conditions in the medium, which were similar to the results observed by other researchers because of the initial concentration of conidial cells or the nutritional imbalance caused by the enormous growth 43. The strain GS115 itself could not synthesize histidine or grow in minimal media without histidine supplementation. High and low levels of histidine affected the enzyme production in the fermentative process. Since GS115 is a His‐deficient strain and pPICZa does not contain a His complement gene, histidine must be added to the medium because GS115 could not synthesize histidine when it was fermented with GS115/pPICZa in industrial medium. Without histidine, the expression was negligible, and the addition of histidine was not sufficient to express the xylanase. When histidine was added at 0.5%, the highest expression was obtained (Figure 1H).

The optimal levels of the eight main factors were selected by a single‐factor test. Because traditional media optimization methods are time consuming and labor intensive, the Plackett–Burman experimental design approach could quickly and efficiently find significant factors among multiple factors 18. Moreover, Plackett–Burman is a common method for statistical screening, and the Plackett–Burman test design had two factor indicators. Therefore, only the factors that had a significant influence on the variables could be selected, and the optimal conditions could not be selected. When the *p* value of the test was less than 0.05, this factor had a significant influence on the variables 39. Therefore, the eight factors were subjected to the Plackett–Burman test. The results showed that the inoculum size, histidine and maize meal had a significant effect on the expression of xylanase by *P. pastoris*.

Laboratory culture media is expensive and inappropriate for large‐scale fermentation. Maize meal and bean pulp are cheap substrates for xylanase production. The high activity of xylanase in the presence of maize meal may be due to the high nutritional content of maize meal, which supports the beginning of microbial growth and replication, and maize meal remains loose even under wet conditions, providing a large surface area for microbial nutrient absorption during SMF. Most studies have found the optimal in carbon, nitrogen, and pH, for the best xylanase activity 42. Agricultural production is a suitable basic substrate for the economic production of xylanases. Wheat bran, corncob, and oat bran have been reported as the most commonly used substrates for xylanase production 40, 41, 44.

However, the Plackett–Burman test method could only find factors that had significant effects, but could not describe the interactions between factors 23. Therefore, this experiment used Plackett–Burman combined with the RSM to find the optimal fermentation conditions. The Box–Behnken design is a type of RSM. It requires less experimentation to measure the interaction between the test factors. The Box–Behnken design is more time saving, labor saving, and effective, and it can effectively analyze the interactions between various factors 31.

The RSM test was an effective method to increase the activity of expression products to reduce costs. The predicted xylanase enzyme activity was estimated at 3236.12 U mL^−1^. To confirm the predicted result, experiments were performed in triplicates using the optimized conditions, and a xylanase activity of 3273 U mL^−1^ was obtained. The experimental value was very consistent with the value of the predicted experiments, which validated the model. After optimization, the activity of xylanase changed from 1550 U mL^−1^ to 3273 U mL^−1^, and the yield of xylanase changed from 0.2784 g L^−1^ to 0.5794 g L^−1^. Compared with the unoptimized level, the activity increased by 2.1 times, an increase of 1.4 times compared to the laboratory medium. The significant increase in the activity of xylanase optimized by the RSM could be due to the significant interactions between the size of the three variables (inoculation, maize meal, and histidine). At present, there is a large amount of literature on the optimization of xylanase using the RSM, but most of the optimized conditions consider chemical media. In this experiment, the medium suitable for large‐scale production, with cornmeal as the carbon source and soybean meal as the nitrogen source, greatly reduced the production cost compare to the original medium, which increased the possibility of the commercial application of xylanase.

SDS–PAGE showed that recombinant xylanase migrated as a single band with the size of 23 kDa (Figure 2). To identify whether the expressed protein was xylanase, the MALDI‐TOF‐MS analysis results showed that the xylanase was produced by fermentation came from *A. niger*. Compared with the original gene, the coverage rate was 78%. The optimized recombinant xylanase gene was identified.

The hydrolysis of the xylanase substrate showed that the hydrolytic products of other substrates were in accordance with previous reports 1. However, the content of X1, X2, and X3 produced by corncob xylan hydrolysis was improved, possibly because corncob as the carbon source of culture medium could induce xylanase to hydrolyze easily.

## 6 CONCLUDING REMARKS

In this work, statistical methods were successfully applied to examine meal and bean pulp as carbon and nitrogen sources, respectively, in the fermentation of GS115/pPICZa‐(XynB‐opt)_2_, and single‐factor experiments combined with Plackett–Burman and Box–Behnken design were used to optimize the fermentation conditions. The optimal concentrations of fermentation components (inoculum size, 3.8%; maize meal, 4.5%; histidine, 0.6%; methanol, 1%; culture volume, 20%; bean pulp, 30 g L^−1^; and Tween‐80, 0.8%; pH, 5.0) were successfully identified. In shake flask experiments through optimization, the best conditions produced 3273 U mL^−1^ xylanase. The statistical methods in this study seemed to be valuable tools optimization which include one‐factor‐at‐a‐time approach, Plackett–Burman design, Box‐Benken design, and Response surface methodology, as the production of xylanase has increased by approximately about 2.1‐fold. Furthermore, crude enzymes had high specific activity against xylan, which indicated that the application of xylanase as feed in industry is suitable.

## AUTHOR CONTRIBUTIONS

Anshan Shan and Ping Yan designed the experiments; Taotao Sun, Ping Yan, Na Zhan, Licong Zhang, and Zhihui Chen were in charge of the main experiments; Taotao Sun, Licong Zhang, and Aizhong Zhang analyzed and processed the data; Taotao Sun and Ping Yan wrote the draft of the manuscript. All authors have read and approved the final manuscript.

## CONFLICT OF INTEREST

The authors have declared no conflict of interest.
